# β-Catenin Drives the FOXC2-Mediated Epithelial–Mesenchymal Transition and Acquisition of Stem Cell Properties

**DOI:** 10.3390/cancers17071114

**Published:** 2025-03-26

**Authors:** Maria Castaneda, Petra den Hollander, Steve Werden, Esmeralda Ramirez-Peña, Suhas V. Vasaikar, Nick A. Kuburich, Claire Gould, Rama Soundararajan, Sendurai A. Mani

**Affiliations:** 1 Department of Translational Molecular Pathology, The University of Texas MD Anderson Cancer Research Center, Houston, TX 77030, USA; maria.castaneda@lilly.com (M.C.); rsoundararajan@mdanderson.org (R.S.); 2 Department of Pathology and Laboratory Medicine, The Warren Alpert Medical School, Brown University, Providence, RI 02912, USA; petra_den_hollander@brown.edu (P.d.H.); nick_kuburich@brown.edu (N.A.K.); claire_gould@brown.edu (C.G.); 3 Legorreta Cancer Center, The Warren Alpert Medical School, Brown University, Providence, RI 02912, USA

**Keywords:** FOXC2, cancer stem cells, β-catenin signaling, epithelial–mesenchymal transition, triple-negative breast cancer, forkhead transcription factors

## Abstract

**Background**: Aggressive forms of breast cancer, such as triple-negative breast cancer (TNBC), are associated with an increase in cancer cells that exhibit stem cell properties. The activation of the epithelial–mesenchymal transition (EMT) program, mediated by the transcription factor FOXC2, generates these stem-like cells. FOXC2 is linked to poor prognoses across various cancer types and is notably upregulated in TNBC, where it establishes and sustains these stem-like cells within the tumor population. **Methods**: Here, we decode the pathways regulating FOXC2 activation using EMT-enriched cell line models. Stemness was assessed using mammosphere assays and mesenchymal markers by western blot. Expression correlations with clinical data was examined using the EMTome. **Results**: We demonstrate that β-catenin serves as a critical mediator of mesenchymal and stemness characteristics through FOXC2 upregulation. By disrupting β-catenin, we find that FOXC2 expression, mesenchymal properties, and stemness are reduced; however, the introduction of exogenous FOXC2 expression in β-catenin deficient cells is enough to restore the mesenchymal and stemness phenotype. These findings support the idea that FOXC2 acts as the downstream regulator of β-catenin and influences both mesenchymal and stemness properties. Moreover, there is a positive correlation between the expression of β-catenin and FOXC2 in various cancer subtypes observed in clinical patient samples. **Conclusions**: Our study clarifies the role of the β-catenin/FOXC2 signaling axis in maintaining stemness properties, suggesting potential targets for TNBC and other cancers driven by EMT-related mesenchymal and stemness characteristics.

## Simple Summary

The most aggressive cancers tend to have a high number of cells that resemble stem cells, which contribute to metastasis, resistance to therapies, and cancer recurrence. The activation of the embryonic program known as epithelial–mesenchymal transition, which is mediated by the transcription factor FOXC2, generates these cancer stem cells. In this study, we demonstrate that WNT/β-catenin signaling, a known inducer of stemness properties, is an upstream regulator of FOXC2 in cells that have undergone EMT. Furthermore, FOXC2 is the downstream effector of β-catenin’s induction of EMT-related mesenchymal and stemness properties. Furthermore, we observed that multiple aggressive cancer types have high levels of β-catenin and FOXC2. Understanding the regulation and activation of FOXC2 during cancer progression will guide the development of therapeutics that can eradicate the cancer stem cell population.

## 1. Introduction

Triple-negative breast cancer (TNBC) is a highly aggressive subtype of breast cancer with a dismal prognosis relative to other breast cancer subtypes. TNBC’s poor prognosis is generally attributed to the ineffectiveness of endocrine therapy and high levels of cancer stem-like cells (CSCs), which fuel recurrence, metastasis, and chemotherapy resistance [1,2,3]. The former is due to the lack or low expression of ER (estrogen receptor), PR (progesterone receptor), and Her2 (human epidermal growth factor 2), which are commonly found in other breast cancer subtypes [3]. Due to the lack of hormone receptors, the clinically established endocrine therapy for other breast cancer types is ineffective on TNBC; thus, TNBC is commonly treated with chemotherapy only. Unfortunately, TNBC is prone to resistance to chemotherapy, leading to the recurrence and progression of the cancer. This resistance is mainly due to the upregulation of the epithelial–mesenchymal transition (EMT) program, leaving TNBC as the most intractable subtype of breast cancer.

The latter TNBC hallmark, the high levels of CSCs, also significantly contributes to the poor prognosis of these patients and is largely attributed to the activation of the EMT program, which generates both mesenchymal and stemness properties in cancer, endowing them with motility, invasiveness, plasticity, and chemotherapy resistance [4,5]. Carcinomas can activate the EMT program in response to a variety of stimuli, including growth factors and cytokines in the tumor microenvironment (i.e., TGF-β1 and IL-6), hypoxia [6], chemotherapy [7], and immune suppressive immune cells [8], inducing mesenchymal and stemness properties. This represents a critical unmet need for TNBC, as the main treatment strategy of chemotherapy is not only resisted by cancers with high levels of EMT signaling, but it also induces EMT, driving increased resistance; thus, identifying targetable vulnerabilities in the EMT program is an unmet need and a promising strategy. We previously demonstrated that FOXC2 is the central transcription factor that induces EMT and stemness through various signaling pathways. CSCs and aggressive metastatic carcinomas express high levels of FOXC2 [9,10,11], which is upregulated downstream of multiple EMT pathways and required for EMT induction [9,10]. Critically, inhibiting FOXC2 by knocking it down or by inhibiting key positive regulators of FOXC2 blocks EMT induction, stemness, and metastasis [9,10,12,13], demonstrating the significance of targeting this critical protein in cancer.

Targeting CSCs will be critical to improving the response of TNBC patients to therapy; however, a lack of understanding of the drivers in the acquisition of this stem-like phenotype has made this challenging. Taking cues from our understanding of FOXC2’s regulation during development can help to determine its role and regulation in cancer progression. FOXC2 knockout mice demonstrated that it is critical in mesenchymal tissue differentiation, specifically for osteoblasts and adipocytes [14]. FOXC2 is required for the differentiation of pre-osteoblast cells into osteoblasts, and FOXC2 overexpression in these cells resulted in increased osteoblast maturation and, interestingly, an upregulation of β-catenin [15]. β-catenin is a critical component of the canonical Wnt signaling pathway, which provides essential regulation of cell proliferation, survival, differentiation, and migration [16]. Subsequent studies further characterized the importance of the FOXC2/β-catenin pathway in differentiation and development. FOXC2 expression is upregulated in mouse muscle stem cells and downregulated during myogenesis [17]. The depletion of FOXC2 in these cells decreases proliferation, while the overexpression of FOXC2 increases proliferation, inhibits differentiation into myotubes, and induces β-catenin signaling [17]. FOXC2 was further validated to induce the Wnt/β-catenin pathway during osteogenic differentiation of bone marrow mesenchymal stem cells by directly binding to and promoting the transcription of *Wnt4*, leading to increased secretion of Wnt4 protein (ligand and agonist of β-catenin signaling) and increased β-catenin signaling [18,19]. Finally, our previous work shows that the exogenous expression of FOXC2 in Madin–Darby canine kidney (MDCK) epithelial cells shifted β-catenin from membrane localization (inactive) to nuclear-localized (active) [9]. Interestingly, there was no increase in β-catenin levels upon exogenous FOXC2 expression [9], a finding that was later supported in pancreatic ductal adenocarcinoma tissues [20]. Together, these studies support that FOXC2 and β-catenin are part of the same signaling network; however, the dynamics and directionality of this signaling network are not fully understood. Critically, the β-catenin/FOXC2 signaling network’s involvement in the EMT-induced gain of mesenchymal and stemness properties, which are essential components in cancer progression and metastasis, have not been characterized.

The WNT/β-catenin (canonical WNT) signaling pathway has been linked to increased CSC activity across various cancer types [21,22,23]. WNT/β-catenin signaling was initially correlated with the generation of CSCs in skin and colon cancer [24,25,26]. Since then, WNT signaling has also been linked with CSC-like phenotype generation, which fuels tumor growth in several other cancers [27]. The activation of the canonical WNT pathway results from the binding of the WNT ligand to the frizzled receptor, which promotes the phosphorylation of β-catenin [28]. Phosphorylation stabilizes β-catenin and results in its nuclear translocation [28]. In the nucleus, β-catenin forms a complex with TCF/LEF, and this complex activates the expression of genes involved in cancer stem cell fate determination [24,29]. β-catenin is stabilized in many cancers, and mutations that lead to constitutive activation of WNT signaling can induce cancer [30]. However, β-catenin activation can occur through WNT-independent mechanisms, and there are reports that β-catenin has TCF/LEF-independent transcriptional functions [31,32]. These alternative signaling pathways regulating β-catenin can lead to nuances and contextual signaling events, especially in cancer.

The EMT-transcription factor, Snail, has also been implicated in WNT/β-catenin signaling. Time-resolved transcriptome analysis upon conditional *Snai1* expression, which encodes Snail, indicated that Snail mediates WNT/β-catenin signaling [33]. Snail acts on the canonical pathway through the upregulation of LEF1, a nuclear binding partner of β-catenin [33]. The inhibition of β-catenin/LEF1 interaction or LEF1 inhibition prevents Snail from inducing β-catenin targets. This demonstrates that Snail is a downstream effector of LEF1/β-catenin and that it is reliant on β-catenin/LEF1’s functional presence to upregulate alternate Wnt/β-catenin target genes associated with EMT-induced invasiveness [33]. In addition, *CTNNB1* (the gene that encodes β-catenin) is a direct transcriptional target of FOXC1, another forkhead box transcription factor, and the closest paralog to FOXC2 (with a 90% full-length sequence identity and a 98% consensus in the forkhead domain) [34,35]. Although FOXC1 and FOXC2 have cooperative roles during development, FOXC2 is directly associated with EMT and the acquisition of CSC properties [36]. To summarize, the β-catenin/FOXC2 signaling network is involved in cellular differentiation and EMT induction. While both proteins are independently associated with an increase of EMT, stemness, and metastasis in cancers, the FOXC2/β-catenin signaling interplay has not been extensively characterized in cancer stemness and mesenchymal traits.

In this study, we examined the role of β-catenin signaling in driving FOXC2-mediated determination of stem-like cells generated by EMT induction. We observed a positive correlation of FOXC2 and nuclear β-catenin in cells that had initiated EMT and in EMT-enriched TNBC cell lines that had high levels of stemness. Disrupting β-catenin in cells that have undergone EMT by constitutive expression of the EMT-transcription factors Snail or Twist leads to a loss of EMT-induced mesenchymal and stemness properties and FOXC2 expression; thus, β-catenin is required for the maintenance of EMT properties and FOXC2 in these cells. Critically, we found that β-catenin induces these mesenchymal and stemness properties directly through FOXC2 upregulation, as exogenous expression of FOXC2 in β-catenin depleted cells restores the lost mesenchymal and stemness properties. Finally, we show a robust positive correlation between FOXC2 and WNT/β-catenin signaling in clinical patient samples based on RNA and protein analysis. Together, our work here identifies the critical role of a β-catenin/FOXC2 signaling axis in EMT, suggesting targets for TNBC and other cancers with EMT-induced mesenchymal and stemness properties.

## 2. Materials and Methods

Cell Culture: All cell lines were cultured at 37 °C in 5% CO_2_. All cell lines derived from immortalized human mammary epithelial cells (HMLE) were cultured as described previously [37]. The HMLE cell line was provided as a gift from Dr. Robert Weinberg, MIT. MCF7 and MDA-MB-231 (ATCC) cells were cultured in DMEM/F12 (Corning, Corning, NY, USA), SUM159 (BioIVT, Westbury, NY, USA) cells were cultured in Ham’s F12 (Corning) medium, HEK293T cell lines were cultured in DMEM (Corning), and all were supplemented with 10% FBS and 1% penicillin and streptomycin.

RNA extraction and qRT-PCR: Total RNA was isolated using the RNeasy kit (Qiagen, Germantown, MD, USA). All quantitative qRT-PCR experiments were run in triplicate, and the comparative Ct method with *RPLPO* as the reference gene was used. The primers used were the following (Millipore Sigma, Burlington, MA, USA): Forward RPLPO, 5′-GCGACCTGGAAGTCCAACTA-3′; reverse RPLPO, 5′-ATCTGCTTGGAGCCCACAT-3′; Forward FOXC2, 5′-GCCTAAGGACCTGGTGAAGC-3′; reverse FOXC2, 5′-TTGACGAAGCACTCGTTGAG-3′; Forward ZEB1, 5′-GCACAACCAAGTGCAGAAGA-3′; and reverse ZEB1, 5′-CATTTGCAGATTGAGGCTGA-3′.

Western Blot: Cells were lysed with RIPA buffer (Sigma, R0278) containing protease and phosphatase inhibitors, and 30–50 μg of protein was denatured in Lamelli sample buffer containing β-mercaptoethanol (BioRad, Hercules, CA, USA #1610747) at 97 °C for 7 min. Samples were loaded onto a 10% SDS-agarose gel and run at 110 V. Antibodies used were anti-Snail (Cell Signaling Technology, Danvers, MA, USA 3879S), anti-FOXC2 (Bethyl Laboratories, Montgomery, TX, USA A302-383A), anti-vimentin (Cell Signaling, 5741S), anti-ZEB1 (Santa Cruz, sc-25388), anti-actin (Santa Cruz Biotechnology, Dallas, TX, USA, sc-1616-R), anti-β-Catenin (BD Biosciences, San Jose, CA, USA, 610153), anti-Fibronectin (BD Biosciences, 1:1000 in 5% milk), and anti-PDGFRβ (Cell Signaling, 2169; 1:1000 in BSA).

Plasmids and Viral Transduction: HMLE-vector, HMLE-Snail, and HMLE-Twist were generated as described previously [4]. Stable cell lines with shβ-Catenin were created through viral transduction. FOXC2 overexpression was performed using lentivirus transduction of the pMIG-FOXC2 vector, as described previously [13].

Immunofluorescence: Cells were plated and after 24 hours cells were fixed in 4% paraformaldehyde. Cells were incubated with primary antibody at 1:100 (FOXC2, R&D, AF 5044) (β-catenin, Cell signaling, 8480) in 1% normal donkey serum at 4 °C overnight. Cells were incubated with a secondary antibody and mounted with DAPI. Fluorescent images of the slides were then taken with a Zeiss microscope at 40× and 60× magnification.

Mammosphere Assay: The mammosphere culture medium was MEGM supplemented with 1% methylcellulose as described previously [4]. Cells were cultured at 500 cells per well in 96-well ultra-low attachment plates in triplicate for 14 days. Every 3 days, 50 µL of fresh media was added. Mammospheres were photographed at 14 days, and spheres with a diameter greater than 80 µm were counted.

Cell-surface Marker Analysis: Cells were harvested and suspended in PBS with 4% FBS. Cells were incubated with anti-CD24 antibody conjugated with PE (BD Biosciences 555428) and anti-CD44 conjugated with APC (BD Biosciences, 559942) and were incubated for 30 min on ice. The cells were washed with PBS and suspended in 200 µL PBS with 4% FBS. The cells were analyzed using a BD Accuri analyzer.

Co-immunoprecipitation Assay: SUM159 cells were cultured in a 10 cm dish until 80% confluency. Cells were washed with PBS, and then 500 µL of cold lysis buffer (20 mM Tris, 137 mM NaCl, and 1% NP-40 containing protease and phosphatase inhibitors) was added. Cells were kept on ice for 30 min and then centrifuged for 20 min at 16,000× *g* at 4 °C. Next, 500 µg protein was incubated with 10 µg of anti-β-catenin antibody (BD Biosciences, 610153) at 4 °C overnight. Subsequently, 100 µL of A/G bead slurry (Thermo Fisher, Waltham, MA, USA, 20421) was added to the solution and incubated for 2 h at room temperature. The solution was spun down for 1 min at 1000× *g*. The supernatant was discarded, and the pellet was washed five times with lysis buffer; 50 µL of Laemmle sample buffer (Bio-Rad, Hercules, CA, USA, 1610747) was added. Samples were boiled at 95 °C for 5 min, and lysates were analyzed by Western blot.

Data analysis: Proteogenomic data of breast cancer patients, such as expression of RNA, Protein/RPPA data for CTNNB1 (β-catenin), ESR1, and FOXC2, were retrieved from Mertins et al.’s dataset [38]. FOXC2 and β-catenin RNA expression analysis was retrieved from the in-house-developed platform EMTome [39]. The dataset consists of TCGA cohort patient genomic, epigenomic, and transcriptomic data for 32 cancer types. The cancer hallmark enrichment score for each patient in a cancer cohort was inferred by the ssGSEA method [39].

Statistical Analysis: Unless otherwise stated, all samples were assayed in triplicate, and all experiments were repeated at least three times. Unless otherwise indicated, data are reported as means ± SD, and significance was calculated using Student’s unpaired two-tailed *t*-test.

## 3. Results

### 3.1. β-Catenin Positively Correlates and Interacts with FOXC2

**Figure 1:**
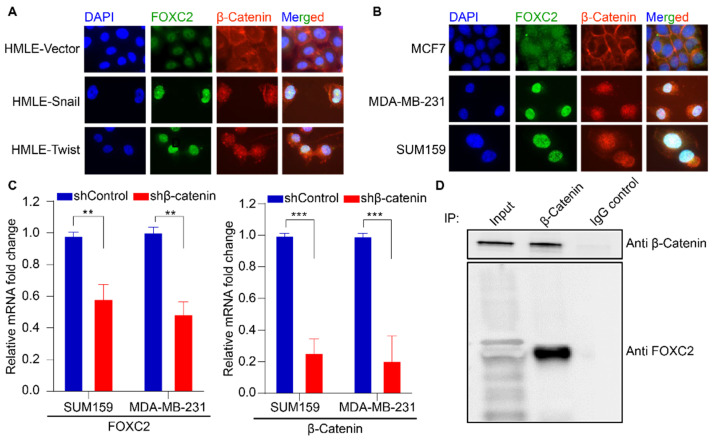
β-catenin and FOXC2 expression are positively correlated and interact in cells with EMT traits. (**A**) Immunofluorescence staining of FOXC2 (green), β-catenin (red), and DAPI (blue) in HMLE cell lines modified to initiate EMT (HMLE-Snail and HMLE-Twist). (**B**) Immunofluorescence staining of FOXC2 (green), β-catenin (red), and DAPI (blue) in human epithelial (MCF7) and TNBC (SUM159 and MDA-MB-231) breast cancer cell lines. (**C**) qRT-PCR quantification of expression levels of *FOXC2* (left) and *CTNNB1* (right) mRNAs in TNBC cell lines with and without treatment with shRNA targeting *CTNNB1* or a control shRNA. (**D**) Western blot of lysates of SUM159 cells without (input) or with *β-catenin* pulldown or incubation with IgG as a control. Statistical comparisons performed by Student’s *t*-test *** *p* < 0.0005, ** *p* < 0.005.

To characterize the FOXC2/β-catenin signaling axis as a function of EMT induction, we performed a co-localization analysis on epithelial cells relative to EMT-induced and enriched epithelial cells. We first used an isogenic system of EMT to characterize this co-localization. Here, we used the HMLE cell lines derived from human mammary epithelial cells that were transduced with a lentiviral vector to overexpress either of the EMT-inducing transcription factors Snail (*Snail1*) or Twist (*Twist1*) to induce and maintain EMT in these cells. We compared these cells to the epithelial HMLE transduced with an empty vector control (pWZL, HMLE-Vector). The HMLE-Snail and HMLE-Twist cells are known to have mesenchymal properties and higher proportions of cells with stemness properties than the control cells. The HMLE cells that express Snail and Twist have basal-like attributes, undergo distinct phenotypic changes upon EMT, and their inherent plasticity allows them to withstand not only EMT but also the manipulation of their gene expression [4,40]. In the HMLE cell model, we observed a co-localization of β-catenin and FOXC2 in the nucleus only upon Snail or Twist expression and not in the HMLE-Vector control (Figure 1A).

We then characterized the co-expression of FOXC2 and β-catenin in breast cancer cell lines with either epithelial or EMT-enriched properties. We used the luminal, estrogen-positive breast cancer cell line MCF7 as an epithelial model, and we used the TNBC cell lines MDA-MB-231 and SUM159 as EMT-enriched models. We observed nuclear localization of both β-catenin and FOXC2 in MDA-MB-231 and SUM159, whereas in the epithelial MCF7, we detected very weak punctate nuclear FOXC2 staining and only cytoplasmic β-catenin staining (Figure 1B). Together, these results suggest that FOXC2 and β-catenin activation (i.e., expression and nuclear localization) is upregulated with EMT.

We then performed qRT-PCR quantification of expression levels of *CTNNB1* and *FOXC2* mRNAs in the TNBC cell lines SUM159 and MDA-MB-231 with or without depletion of β-catenin using shRNA-mediated knockdown. *FOXC2* expression levels were reduced upon β-catenin knockdown (Figure 1C), suggesting that FOXC2 expression in the EMT-enriched cancer cells is dependent on β-catenin. This observation is interesting as most of the reported FOXC2/β-catenin signaling networks from several studies describe the role of FOXC2 in inducing β-catenin expression rather than FOXC2 being induced by β-catenin [15,16,17,18,19,20].

Having determined the co-localization of β-catenin and FOXC2, as well as their correlative expression in TNBC cell lines, we sought to determine whether FOXC2 and β-catenin physically interact. We immunoprecipitated β-catenin from SUM159 cells and performed a Western blot for FOXC2 in the precipitate. We observed that FOXC2 physically interacts with β-catenin (Figure 1D). Collectively, these data demonstrate a clear positive correlation between β-catenin and FOXC2 expression, that these two proteins interact, and that FOXC2’s expression is dependent on β-catenin.

### 3.2. β-Catenin Mediates FOXC2-Associated EMT and Acquisition of Stem Cell-like Properties

**Figure 2:**
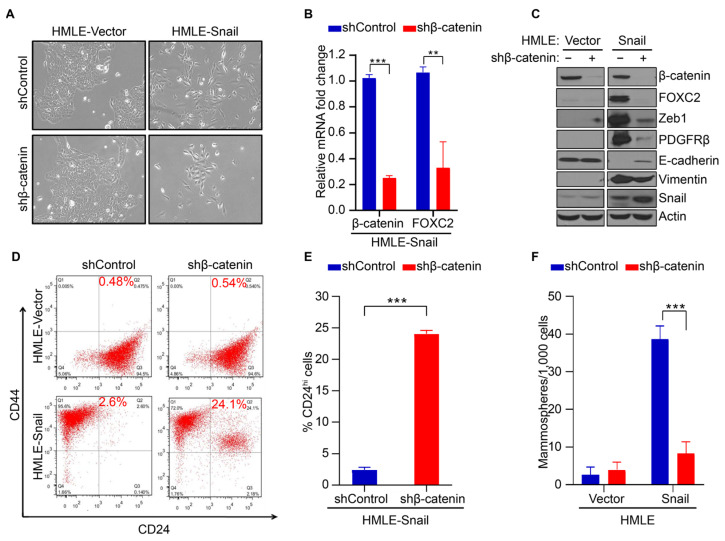
β-Catenin mediates FOXC2-associated EMT and acquisition of stem cell-like properties. (**A**) Phase contrast images of HMLE-Vector (left) and HMLE-Snail (right) cells treated with a control shRNA (top row, shControl) or a shRNA targeting *CTNNB1* (bottom row, shβ-catenin). (**B**) qRT-PCR quantification of expression levels of *CTNNB1* and *FOXC2* in HMLE-Snail cells expressing indicated shRNA. (**C**) Western blot analysis of HMLE-Vector (left) and HMLE-Snail (right) cell lines for mesenchymal markers after transducing with shControl or shβ-catenin. (**D**) FACS analysis of CD44 and CD24 expression in HMLE-Vector (top) and HMLE-Snail (bottom) after transducing with shControl or shβ-catenin. (**E**) FACS quantification of CD24^high^ cells in HMLE-Snail cells with or without β-catenin knockdown. (**F**) Quantification of mammospheres per 1000 cells in HMLE-Vector and HMLE-Snail cells with or without β-catenin knockdown. Error bars represent s.d. from triplicate experiments. Statistical comparisons were performed by Student’s *t*-test. *** *p* < 0.0005; ** *p* < 0.005.

For the remainder of the study, we chose the model systems of HMLE-Snail and HMLE-Twist as well-characterized representatives of EMT attributes in mammary cells to study the relationship between the FOXC2/β-catenin signaling axis and the mesenchymal and stem-like properties that are induced during EMT [4]. We initiated the studies through the knockdown of β-catenin (HMLE-Snail shβ-catenin and HMLE-TWIST shβ-catenin) and used a non-targeting shRNA for control (shControl). The HMLE-Snail and HMLE-Twist cell lines have a mesenchymal-like morphology (Figure 2A and Figure S1A). The depletion of β-catenin was sufficient to shift the morphology from mesenchymal-like to a more epithelial-like morphology, such as enhanced cell–cell contact, growth in clusters, and a more cuboidal phenotype (Figure 2A and Figure S1A). This indicates the reversal of EMT. In addition, the levels of *FOXC2* mRNA were significantly reduced after β-catenin knockdown (Figure 2B and Figure S1B), mirroring our results in the TNBC cell lines SUM159 and MDA-MB-231 (Figure 1C).

To determine whether depletion of β-catenin reduced levels of FOXC2 protein and inhibited EMT, we analyzed levels of FOXC2 and other epithelial and mesenchymal markers. For mesenchymal markers, we used ZEB1 (a well-described EMT-transcription factor) [41], PDGFRβ (a platelet-derived growth factor receptor beta, which is upregulated during EMT [10,42]), and vimentin (an intermediate filament and a well-described marker of EMT). We used E-cadherin (an adhesion junction protein expressed in epithelial cells) [43,44,45,46] as an epithelial marker. The depletion of β-catenin inhibited EMT, as demonstrated by the reduction in mesenchymal markers and the simultaneous induction of E-cadherin (Figure 2C and Figure S1C), in agreement with previous reports [47,48]. Together, these data support that β-catenin is required for the maintenance of mesenchymal properties following EMT, and the loss of β-catenin results in the reversion to an epithelial phenotype in cells that have undergone EMT.

Next, we tested whether β-catenin knockdown decreases another critical product of EMT, namely stemness properties [4]. We first characterized the impact of knocking down β-catenin on CD44/CD24 expression, where the CD44^hi^/CD24^low^ configuration is a positive marker for human breast CSCs and normal mammary epithelial stem cells [4,49,50]. EMT induction leads to the generation of CD44^hi^/CD24^low^ cells with stemness properties [4]. As expected, we did not observe a CD44^hi^/CD24^low^ population in HMLE-vector cells but did see a robust CD44^hi^/CD24^low^ population following EMT induction in HMLE-Snail and HMLE-Twist cells (Figure 2D and Figure S1D). There were no significant changes in the CD44^hi^/CD24^low^ populations in HMLE-vector cells following the depletion of β-catenin; however, knocking down β-catenin led to a robust increase in the number of CD24^hi^ (more differentiated) cells in HMLE-Snail and HMLE-Twist cells (a 9.3-fold and 13-fold increase, respectively) (Figure 2D,E and Figure S1D,E). In order to validate that the observed decrease in CD44^high^/CD24^low^ cells is reflective of a reduction in stem cell-like properties, we performed a mammosphere formation assay, an established method for measuring functional stemness properties [4,51,52,53]. Upon EMT initiation by induction of Snail or Twist expression, the number of mammospheres increased dramatically (Figure 2F and Figure S1F). The number of mammospheres was significantly reduced in HMLE-Snail and HMLE-Twist cell lines that are depleted with β-catenin. Together, these data support that the mesenchymal and stemness properties endowed through EMT require β-catenin, with a dramatic loss of FOXC2 mRNA and protein levels following the depletion of β-catenin (Figure 2B,C and Figure S1B,C).

### 3.3. Expression of FOXC2 in the Absence of β-Catenin Restores EMT and Stem Cell Properties

**Figure 3:**
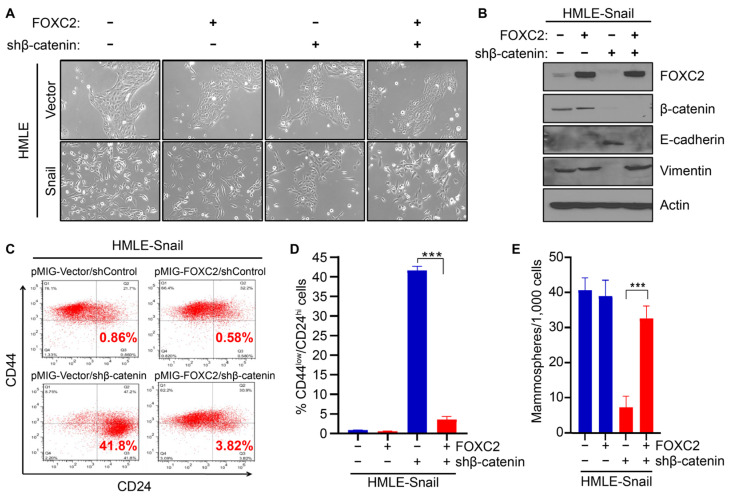
Exogenous expression of FOXC2 restores mesenchymal and stem cell-like properties in the absence of β-catenin. (**A**) Phase contrast images of HMLE-Vector (top) and HMLE-Snail (bottom) cells with or without *CTNNB1* knockdown and with or without exogenous FOXC2 expression. (**B**) Western blot analysis for mesenchymal markers in HMLE-Snail cells with or without *β-catenin* knockdown and with or without exogenous FOXC2 expression. (**C**) FACS analysis of HMLE-Snail cell lines with or without *CTNNB1* knockdown and with or without exogenous FOXC2 expression. (**D**) Quantification of CD44^low^/CD24^high^ (differentiated, non-stem-like) levels in HMLE-Snail cells with or without *CTNNB1* knockdown and with or without exogenous FOXC2 expression. (**E**) Quantification of mammosphere-forming capacity per 1000 cells in control (left) and HMLE-Snail (right) cells with or without *CTNNB1* knockdown and with or without exogenous FOXC2 expression. Error bars represent standard deviation from triplicate experiments. Statistical comparisons were performed by Student’s *t*-test. *** *p* < 0.0005.

Having established a direct correlation between FOXC2 and β-catenin levels, with the decrease in β-catenin leading to a downregulation of FOXC2 and the loss of the corresponding EMT and stemness properties, we next characterized the order of the FOXC2/β-catenin signaling pathway. Specifically, we tested the hypothesis that FOXC2, whose expression is lost upon β-catenin knockdown (Figure 1C and Figure 2B,C), is downstream of β-catenin’s mesenchymal and stemness enforcing signaling. For this, we utilized HMLE-Vector, HMLE-Snail, and HMLE-Twist cell lines transduced with either shControl or shβ-catenin that were then transduced with either pMIG-FOXC2 or pMIG-empty vector. Knockdown of β-catenin induces an epithelial phenotype in EMT-enriched cells; however, exogenous expression of FOXC2 was sufficient to rescue the lost mesenchymal-like morphology (Figure 3A and Figure S2A). Western blot analysis demonstrated that exogenous FOXC2 expression resulted in an increase in vimentin protein levels (mesenchymal marker) and a decrease in E-cadherin protein levels (epithelial marker) regardless of whether β-catenin was present or depleted (Figure 3B and Figure S3B). These data suggest that FOXC2 expression is sufficient to rescue the loss of the mesenchymal phenotype because of β-catenin depletion in cells that had undergone EMT.

We then asked whether the rescue of the mesenchymal phenotype by exogenous expression of FOXC2 was accompanied by a restoration of the stemness phenotype. Again, we observed that the depletion of β-catenin in cells constitutively expressing Snail or Twist led to a loss of the CD44^hi^/CD24^low^ stem-like population with a gain in the CD44^low^/CD24^hi^ population that represents differentiated cells (Figure 3C,D and Figure S3C,D). Upon FOXC2 exogenous expression, we observed a restoration of the CD44^hi^/CD24^low^ stem-like population and a reduction in the CD44^low^/CD24^hi^ differentiated population for both HMLE-Snail and HMLE-Twist cell lines depleted of β-catenin (Figure 3C,D and Figure S3C,D). Specifically, we observed a 7.1-fold increase of the CD44^hi^/CD24^low^ stem-like population in HMLE-Snail shβ-catenin and a 2-fold increase in HMLE-Twist shβ-catenin cells after exogenous expression of FOXC2. Likewise, the expression of FOXC2 restored the mammosphere-forming ability of HMLE-Snail and HMLE-Twist cells that were depleted of β-catenin (Figure 3E and Figure S2E). Collectively, these data support our hypothesis that FOXC2 is downstream of β-catenin; however, we saw that exogenously expressing FOXC2 bypasses the loss of β-catenin and is sufficient to induce and maintain the mesenchymal and stem-like phenotype granted by EMT, suggesting that β-catenin’s EMT-maintaining properties depend on and work through FOXC2 specifically.

### 3.4. β-Catenin and FOXC2 Are Directly Correlated in Clinical Datasets of Cancer Patients

Thus far, we have demonstrated the importance of the β-catenin/FOXC2 signaling pathway in maintaining EMT-induced mesenchymal and stemness programs. Upregulation of EMT in carcinoma patients has long been an established phenotype of many aggressive cancers [54]. To understand the clinical implications of the β-catenin/FOXC2 signaling pathway, we examined the EMTome, a public resource for cancer dataset analysis, to assess the correlation between FOXC2 and β-catenin in cancer patients across various carcinoma types [39]. Both FOXC2 and β-catenin expression are associated with upregulation of the hallmark TGFβ, EMT, WNT/β-catenin, EMT, and Notch signaling (Figure S3). These pathways are involved in the regulation of CSC properties [21,27]. In addition, both FOXC2 and β-catenin hallmark gene sets are correlated with the downregulation of MYC targets, DNA repair, and oxidative phosphorylation (Figure S3), which are known characteristics of cancer progression [55,56,57]. Interestingly, these correlations were conserved across various other cancer types, including prostate, colon, and ovarian cancer.

**Figure 4:**
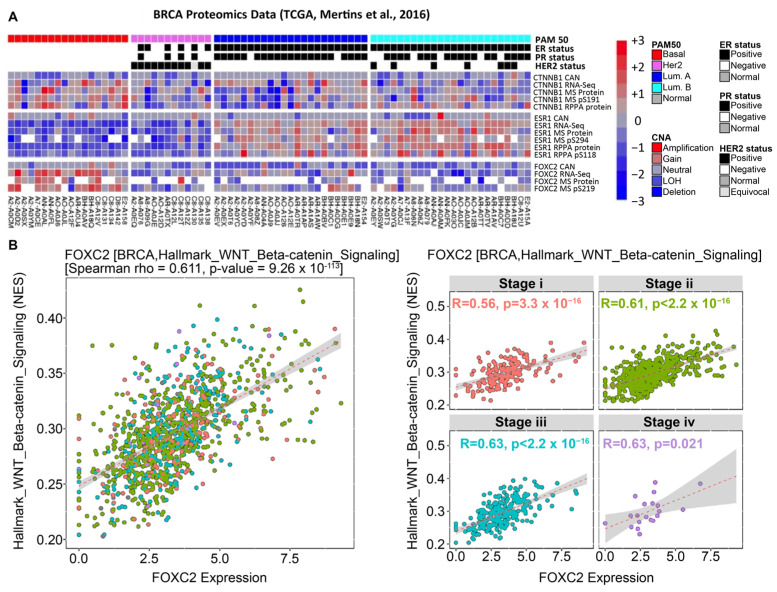
β-catenin and FOXC2 are correlated in clinical samples [38]. (**A**) TCGA data of breast cancer subtypes vs. expression of RNA, Protein/RPPA data for CTNNB1 (β-catenin), ESR1, and FOXC2. FOXC2 and β-catenin are highly expressed in basal breast cancer subtypes as compared with those in Her2 or luminal breast cancer subtypes. (**B**) EMTome generated data for correlation of Wnt/β-catenin signaling and FOXC2 expression during different stages in breast invasive carcinoma.

Next, we analyzed proteomic and RNA-sequencing data from the TCGA, examining breast cancer specifically [38]. The analysis of TCGA data demonstrates a direct correlation between β-catenin expression and FOXC2 expression, with an exceptionally high correlation in basal breast cancers (Figure 4A). About 70% of TNBCs are of the basal-like subtype, and basal-like cancers have higher CSC capabilities than the luminal breast cancer subtype [2,58]. We then performed an EMTome correlative study examining the expression of WNT/β-catenin and FOXC2 across invasive breast carcinoma subtypes. This analysis supported a positive correlation between these two pathways (Figure 4B). Collectively, the TCGA and EMTome data extend and further support the notion that FOXC2 and β-catenin are predominantly co-active in aggressive carcinomas in patient samples. Our findings demonstrate the importance of the β-catenin/FOXC2 signaling axis in maintaining the EMT-induced mesenchymal and stem-like properties, and the co-expression of these EMT-regulating proteins is clinically relevant in patient samples. These findings highlight the in-depth involvement of β-catenin/FOXC2 in aggressive carcinomas and further encourage the research and development of targetable vulnerabilities in this signaling nexus.

## 4. Discussion

**Figure 5:**
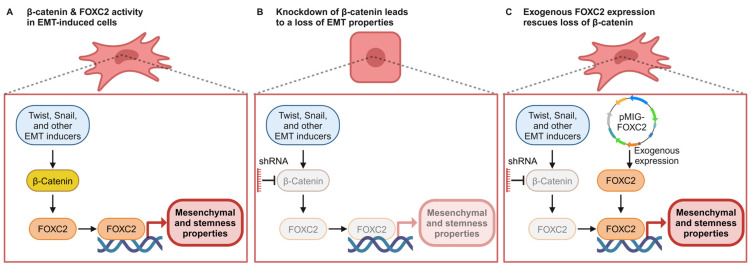
Schematic depiction of β-catenin/FOXC2 signaling and expression on the mesenchymal and stemness properties in cells enriched for EMT traits. (**A**) β-catenin signaling is correlated with the expression of FOXC2 in TNBC cells and mammary cells with stemness properties, whereas FOXC2 levels in cells that have undergone EMT are reliant on β-catenin. (**B**) shRNA-mediated depletion of β-catenin leads to a decrease in FOXC2 expression and the loss of mesenchymal and stem-like properties. (**C**) In the absence of β-catenin due to shRNA-mediated inhibition, exogenous FOXC2 expression is sufficient to restore the lost mesenchymal and stem-like properties in cells that have undergone EMT.

There is a distinct and notable correlation between upregulated β-catenin signaling and the acquisition of stem-like capabilities across various cancer types [59]. Here, we demonstrate that the EMT master regulator, FOXC2, is a downstream target of the β-catenin pathway (Figure 5A) and the key mediator of β-catenin-facilitated stemness and mesenchymal properties in cells that have initiated EMT. Supporting this, a prior developmental biology manuscript reported that β-catenin could increase the expression of FOXC1 and FOXC2 [60]. However, this study was focused on cellular differentiation during mitogenesis, while our study is focused on understanding the role of FOXC2/β-catenin in EMT, which is implicated in cancer progression and metastasis. The EMT-induced and enriched cells lost mesenchymal and stemness properties following the depletion of β-catenin, along with the downregulation of FOXC2 expression (Figure 5B). It is critical to understand the HMLE-Snail and HMLE-Twist system in the interpretation of these results. Snail and Twist are strong inducers of the EMT program, and these HMLE cells have exogenous and constitutive expression of these transcription factors [4]. Despite this strong induction of the EMT pathway, disrupting β-catenin was sufficient to override the consecutive expression of Snail and Twist, suggesting that the EMT-induced mesenchymal and stemness properties by Snail and Twist rely on β-catenin. However, we observed a rescue of the lost mesenchymal and stemness properties in HMLE-Snail/Twist-shβ-catenin cells upon exogenous FOXC2 expression (Figure 5C). This implies that the EMT-derived mesenchymal and stemness properties from the overexpression of Snail or Twist rely not just on β-catenin but on FOXC2 as well. Additionally, this finding supports that FOXC2 is required for the mesenchymal and stemness traits obtained through EMT and β-catenin signaling. In other words, FOXC2 is a convergence point of multiple EMT induction pathways and for β-catenin’s induction of mesenchymal and stemness traits, and it would be interesting to modulate the β-catenin-FOXC2 axis in vivo to test its role in chemoresistance and metastasis.

Multiple reports have described that FOXC2 functions upstream of β-catenin, leading to its induction [15,16,17,18,19,20]. While our work here does not contradict these prior findings, there is likely a positive feedback loop in cells that have undergone EMT where FOXC2 directly or indirectly induces β-catenin and vice versa. More work is needed to characterize this putative positive feedback loop. GeneHancer analysis identifies LEF1 and TCF binding sites (the DNA binding transcription factors in the activated β-catenin complex) in the *FOXC2* promoter region [61], supporting our findings and suggesting that β-catenin directly upregulates *FOXC2* transcription. Interestingly, we did not see FOXC2 binding sites in the promoter region of *CTNNB1* (the gene that encodes β-catenin). However, these findings do not conclude that in our models of EMT that FOXC2’s activation by β-catenin occurs through WNT signaling or relies on LEF/TCF-mediated gene transcription [31,32]. More work will be needed to characterize the upstream signaling pathways and the transcription factors that regulate the β-catenin/FOXC2 signaling axis described here.

Additionally, in our prior work that established the role of FOXC2 in carcinoma progression through the induction of EMT, we did not see β-catenin levels significantly change as a result of FOXC2 expression [9], an observation supported in PDAC [20]. Instead, the exogenous expression of FOXC2 in Madin–Darby canine kidney (MDCK) epithelial cells caused β-catenin localization to shift from membrane-bound (inactivated) to nuclear-localized (activated) [9]. While more work is required to test this, FOXC2 is suggested to be an indirect activator of β-catenin’s nuclear localization and activation rather than directly regulating its transcription.

EMT induction leads to the acquisition of CSCs, which have plasticity, are often chemo-resistant, and induce metastases [62,63,64,65,66]. FOXC2 mediates the initiation of EMT as well as the induction and maintenance of the stem cell-like phenotype [10]. The link between FOXC2 and β-catenin has been previously shown by analysis of osteoblast differentiation, and β-catenin and FOXC2 have been individually linked to stemness trait acquisition in various cancers, including breast cancer [22,29,65]. However, we extended this previous work to demonstrate that β-catenin and FOXC2 interact directly, β-catenin is required for EMT maintenance in cells that have undergone EMT, and FOXC2 is required for β-catenin’s EMT signaling (Figure 5A). We showed this in epithelial cells engineered to undergo EMT as well as in established human TNBC cell lines. Validation of the nuclear localization of β-catenin and FOXC2 and this signaling axis in TNBC tissues with EMT enrichment would be of clinical interest.

We created HMLE lines that express shRNA targeting *CTNNB1* (the gene that encodes β-catenin) in HMLE-Vector (epithelial) and HMLE that was induced to undergo EMT (HMLE-Snail and HMLE-Twist). Interestingly, the ability of exogenous FOXC2 to rescue the loss of an EMT phenotype in cells depleted of β-catenin suggests that the EMT-induced mesenchymal and stem-like phenotypes that β-catenin maintains work through and require FOXC2 expression. FOXC2 was shown to interact with β-catenin in the context of lymphatic vasculature upon drug treatment that enhanced β-catenin signaling [66] and in PDAC cells [20]; however, here we report that this interaction occurs in cells that have undergone EMT. While we observed nuclear co-localization and interaction between FOXC2 and β-catenin, it appears that FOXC2 does not have to bind with β-catenin to activate genes responsible for mesenchymal and stemness properties since exogenous FOXC2 was able to restore these programs even when β-catenin is depleted (Figure 5C). More research will be needed to fully describe the FOXC2/β-catenin signaling network. For example, what is the functional transcriptional regulation of the β-catenin/FOXC2 complex, and how are these gene targets changed when β-catenin is depleted and unable to form this complex? Furthermore, research is needed to determine putative targetable vulnerabilities in this pathway that could be used for translational studies.

## 5. Conclusions

We have shown that β-catenin and FOXC2 co-localize in the nuclei of cells that have undergone EMT in human epithelial cells and TNBC cell lines. The two proteins interact directly, as demonstrated by co-immunoprecipitation. Furthermore, although the depletion of β-catenin led to a decrease in both mesenchymal and stemness properties, the effects of β-catenin loss were mitigated by FOXC2 overexpression. Thus, we conclude that β-catenin signaling serves as an upstream regulator of FOXC2 activity (Figure 5A); β-catenin promotes EMT through FOXC2-mediated mesenchymal and stemness programs (Figure 5B), and the expression of FOXC2 is sufficient to activate these programs in the absence of β-catenin, implying that β-catenin’s EMT activity operates specifically through FOXC2 (Figure 5C). These findings suggest that for breast cancers exhibiting elevated β-catenin signaling, therapies targeting FOXC2 should be explored to eliminate the stem-like subpopulation, leading to reduced chemotherapy resistance and metastasis. 

## Data Availability

The data generated or analyzed during this study are available from the corresponding author upon reasonable request.

## Supplementary Materials

The following supporting information can be downloaded at https://www.mdpi.com/article/10.3390/cancers17071114/s1, Figure S1: β-Catenin mediates FOXC2-associated EMT and acquisition of stem cell-like properties in other cell models of EMT induction; Figure S2: Exogenous expression of FOXC2 restores mesenchymal and stem cell-like properties in other cell models of EMT induction in the absence of β-catenin; Figure S3: β-catenin and FOXC2 signaling pathways correlation.
