# Supplementary material for: β-Catenin Drives the FOXC2-Mediated Epithelial–Mesenchymal Transition and Acquisition of Stem Cell Properties
Source: Cancers (Basel). 2025 Mar 26;17(7):1114. doi: 10.3390/cancers17071114 (PMC11987759; doi:10.3390/cancers17071114)
Supplement: Supplementary file 1 [file cancers-17-01114-s001.zip › cancers-3320671-supplementary.pdf]

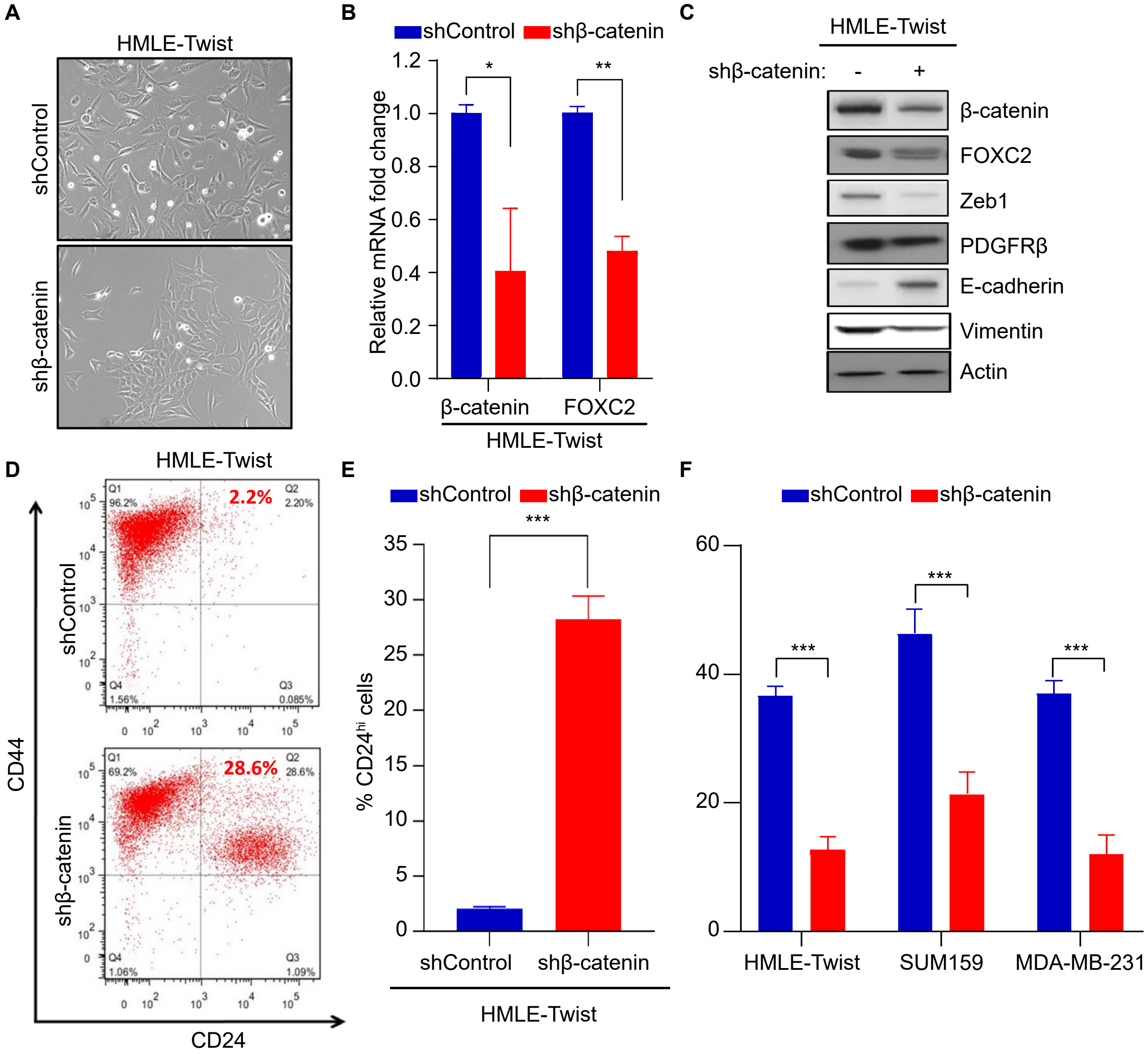

Supplemental Figure S1: β-Catenin mediates FOXC2-associated EMT and acquisition of stem cell-like properties in other cell models of EMT induction. A) Phase contrast images of HMLE-Twist (right) cells treated with a control shRNA (top) or a shRNA targeting *β-catenin* (bottom). **B)** qRT-PCR quantification of expression levels of *β-catenin* and *FOXC2* in HMLE-Twist cells treated with indicated shRNA. **C)** Western blot analysis of HMLE-Twist cells for mesenchymal markers after transducing with shControl or shβ-catenin. **D)** FACS analysis of CD44 and CD24 expression in HMLE-Twist after transducing with shControl or shβ-catenin. **E)** FACS quantification of $CD24^{high}$ cells in HMLE-Twist cells with or without β-catenin knockdown. **F)** Quantification of mammospheres per 1000 cells in HMLE-Twist cells and in TNBC cell lines enriched with EMT properties (SUM159 and MDA-MB-231) with or without β-catenin knockdown. Error bars represent s.d. from triplicate experiments. Statistical comparisons were performed by Student's t-test. *$p<0.05$; ***$p<0.0005$; **$p<0.005$.

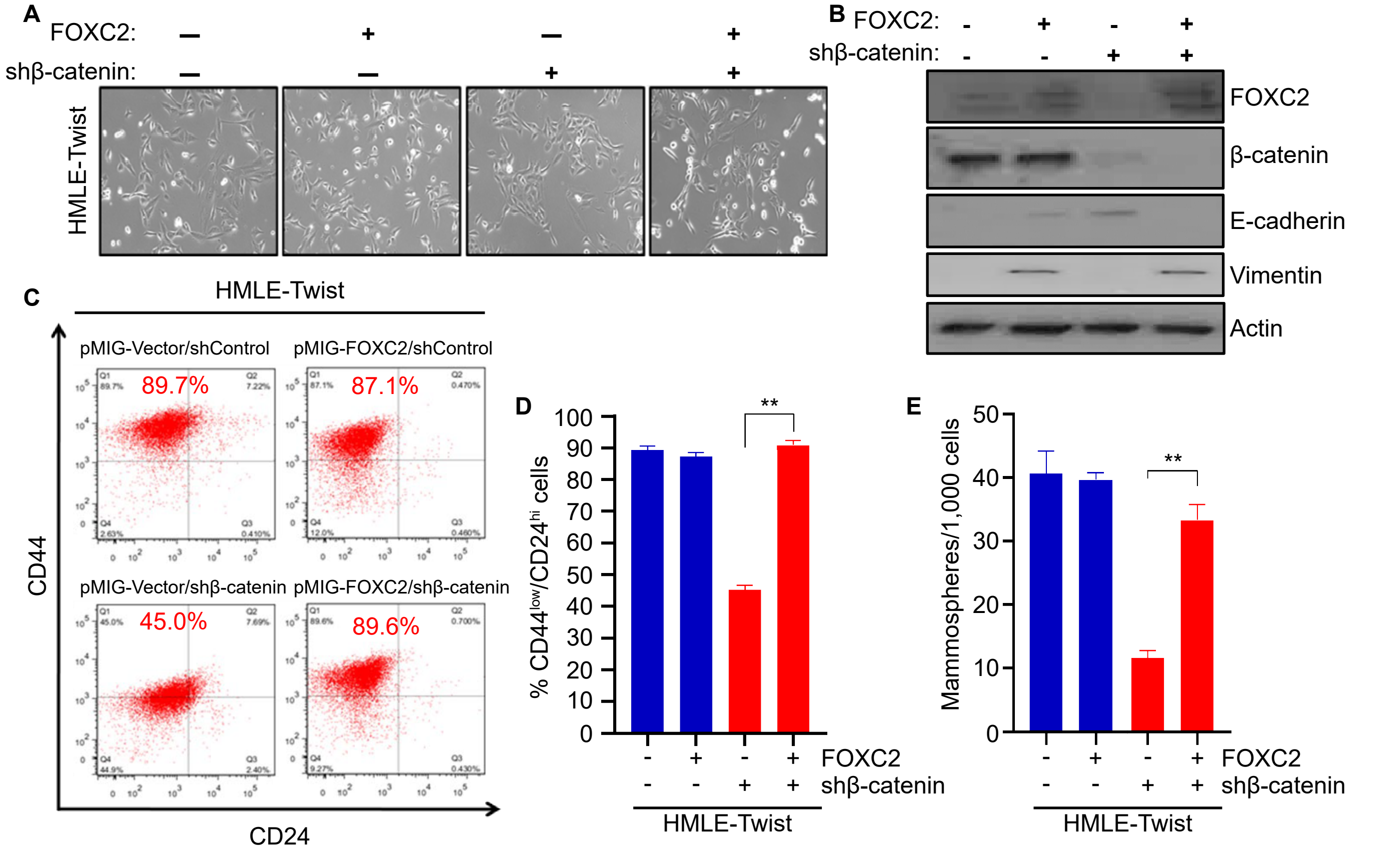

Supplemental Figure S2: Exogenous expression of FOXC2 restores mesenchymal and stem cell-like properties in other cell models of EMT induction in the absence of β-catenin. A) Phase contrast images of HMLE-Twist with or without *β-catenin* knockdown and with or without exogenous FOXC2 expression. **B)** Western blot analysis for mesenchymal markers in HMLE-Twist cells with or without *β-catenin* knockdown and with or without exogenous FOXC2 expression. **C)** FACS analysis of HMLE-Twist cell lines with or without *β-catenin* knockdown and with or without exogenous FOXC2 expression. **D)** Quantification of $CD44^{low}/CD24^{high}$ (differentiated, non-stem like) levels in HMLE-Twist cells with or without *β-catenin* knockdown and with or without exogenous FOXC2 expression. **E)** Quantification of mammosphere-forming capacity per 1000 cells in HMLE-Twist cells with or without *β-catenin* knockdown and with or without exogenous FOXC2 expression. Error bars represent standard deviation from triplicate experiments. Statistical comparisons were performed by Student's t-test. ***p<0.005; ***p<0.0005.

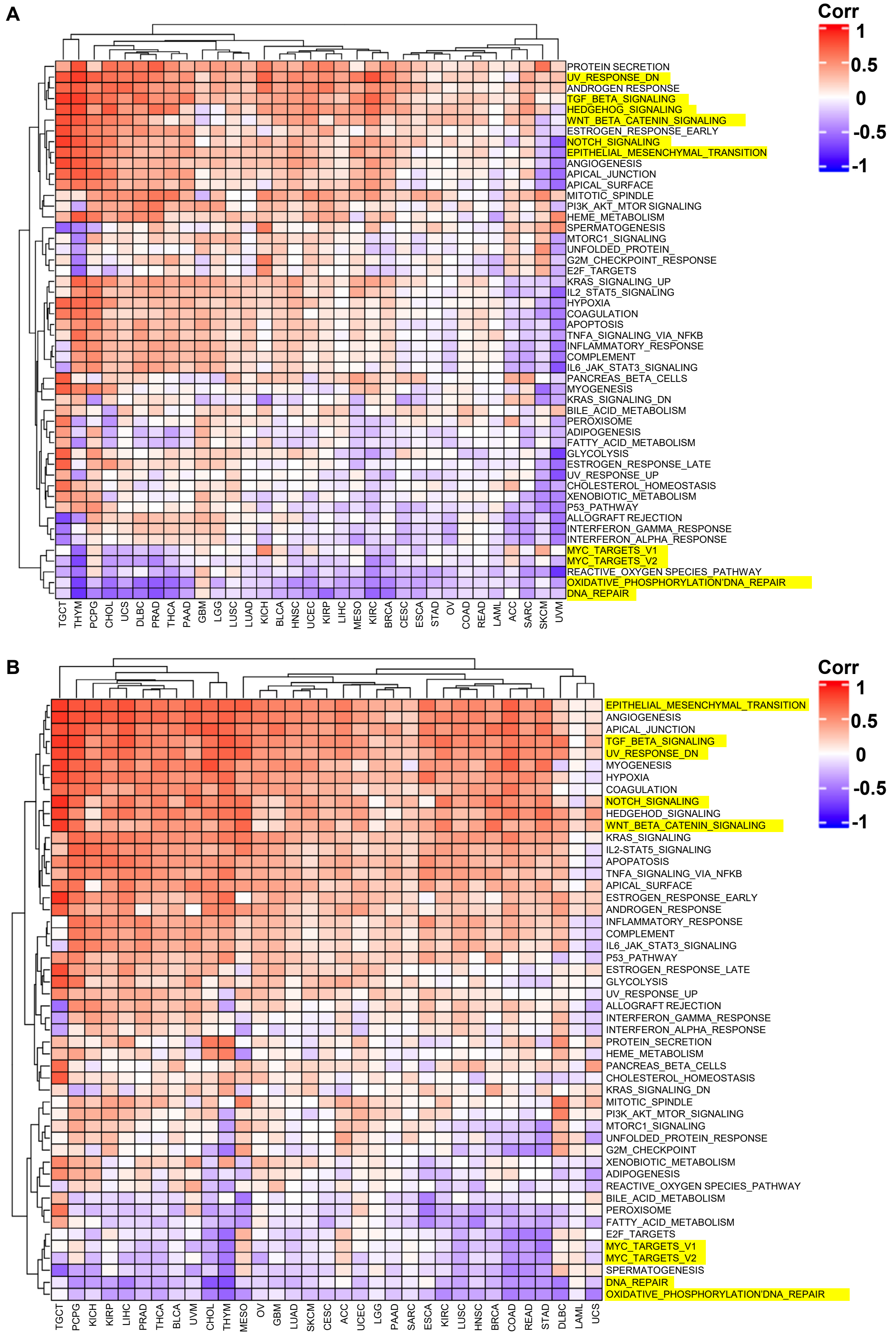

Supplemental Figure S3: β-catenin and FOXC2 signaling pathways correlation A) EMTome generated data for mediated signaling pathways by B-catenin (top) and FOXC2 (bottom) in various cancer subtypes. Highlighted regions indicate similarly regulated pathways, with both positively correlating with UV response, NOTCH signaling, and TGFB signaling, among others.

IB: anti-β-catenin on β-catenin coIP

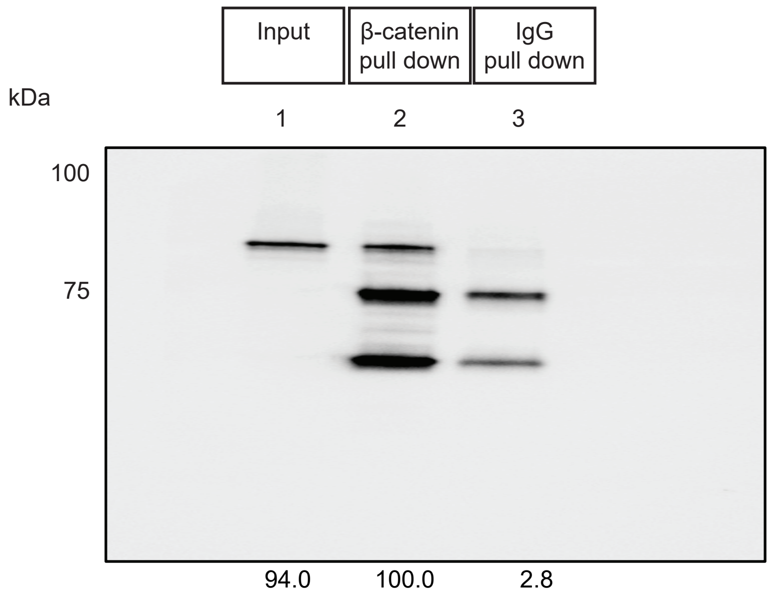

IB: anti-FOXC2 on β-catenin coIP

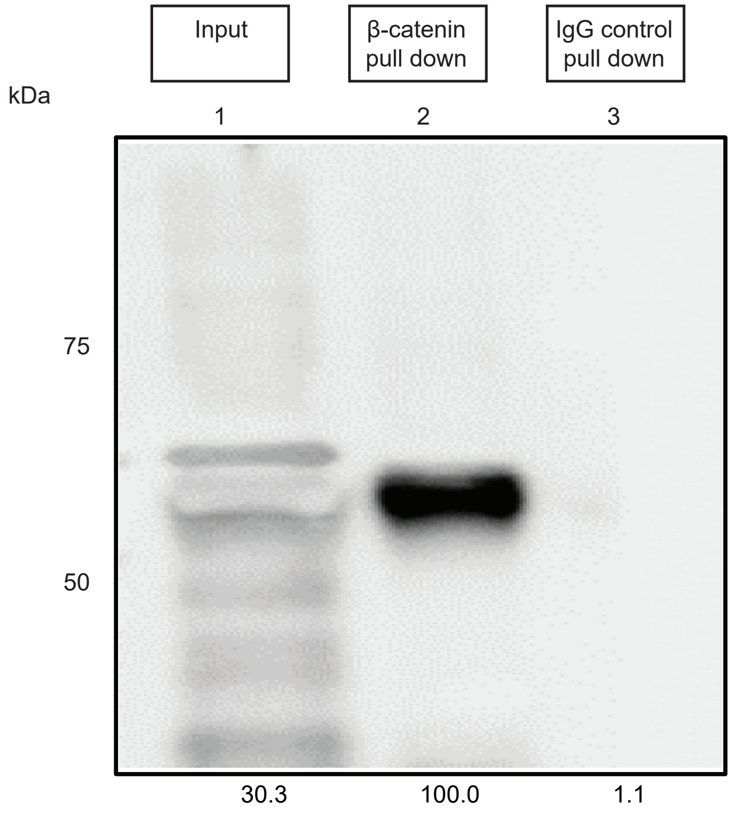

IB: anti-β-catenin

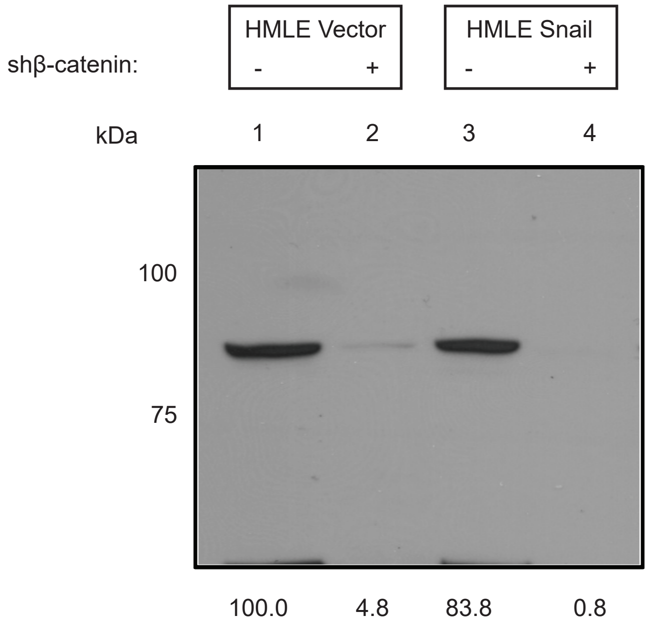

IB: anti-FOXC2

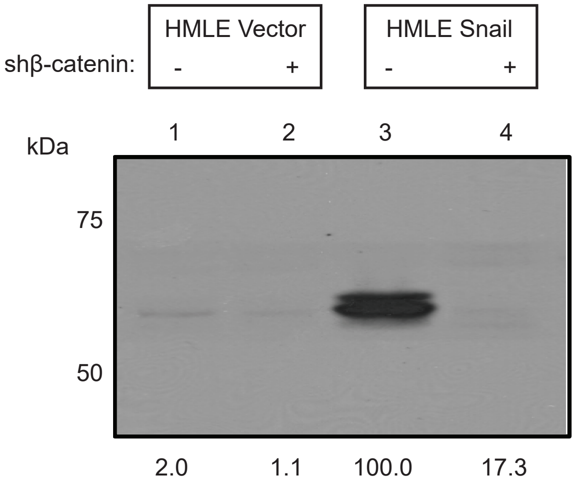

IB: anti-ZEB1

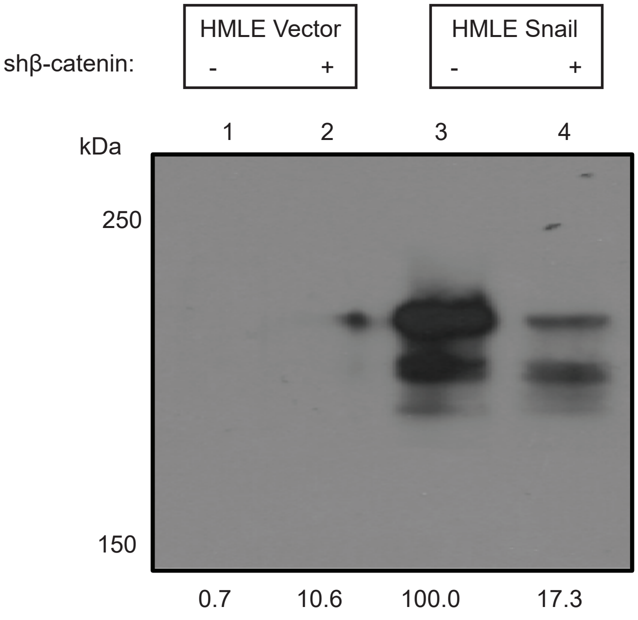

IB: anti-PDGFRβ

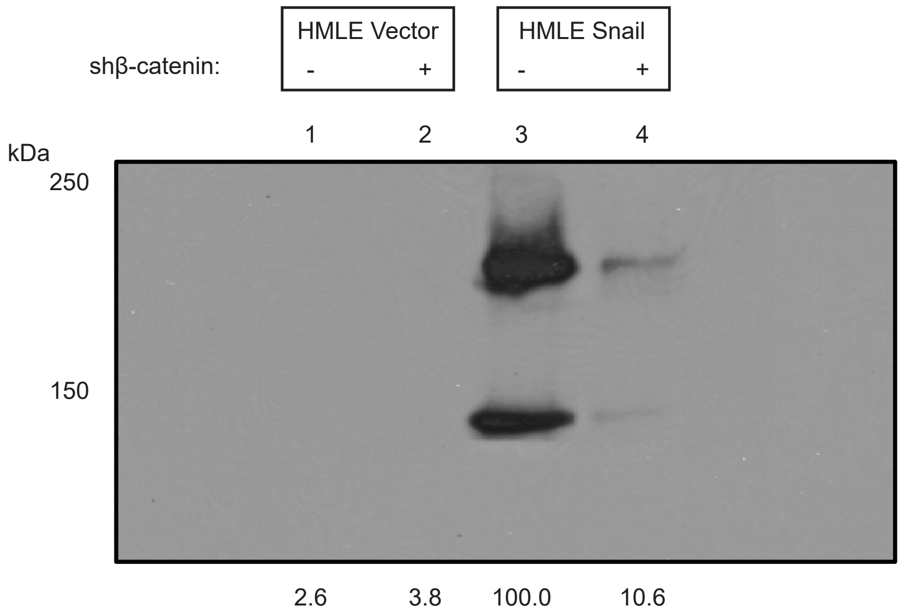

IB: anti-E-cadherin

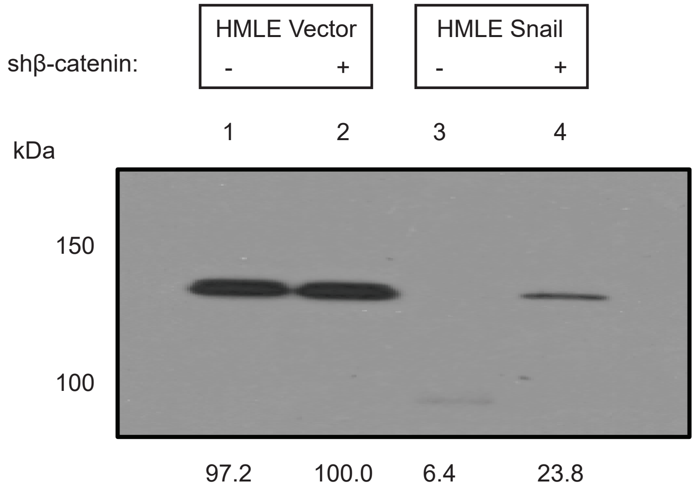

IB: anti-Vimentin

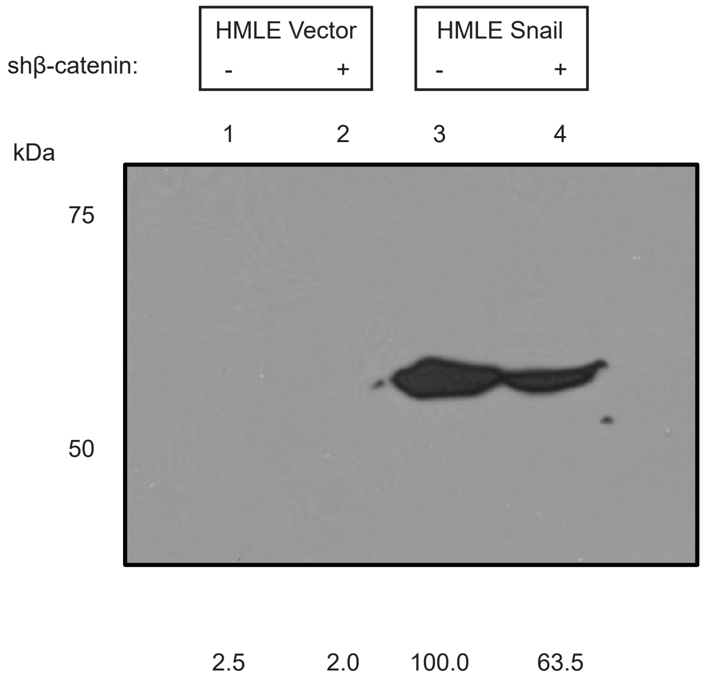

IB: anti-Snail

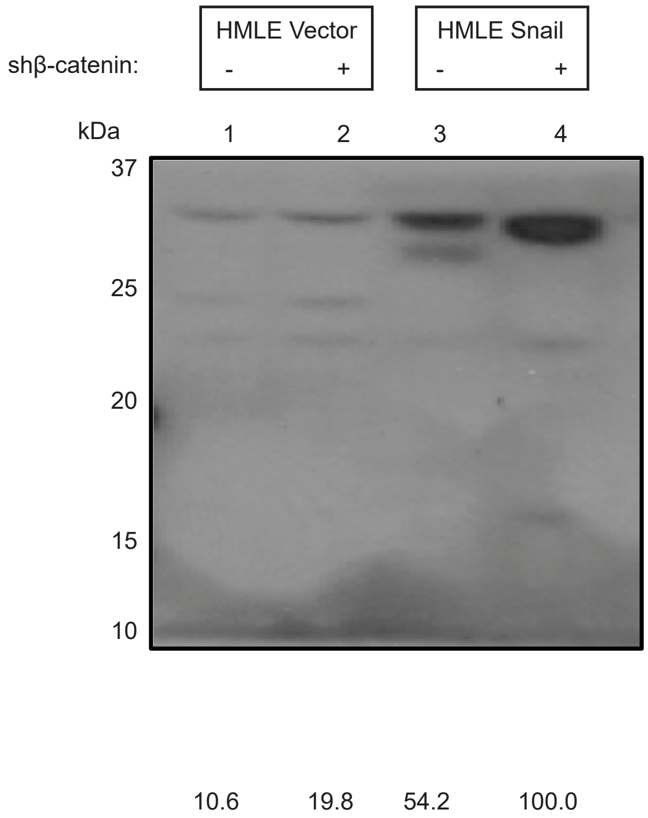

IB: anti-Actin

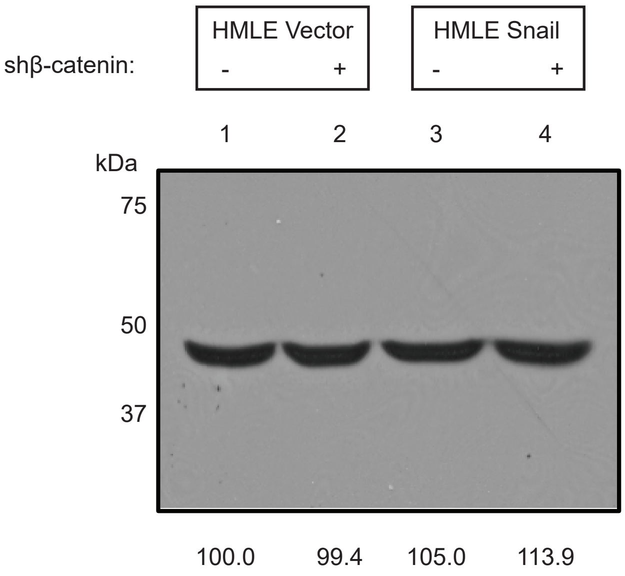

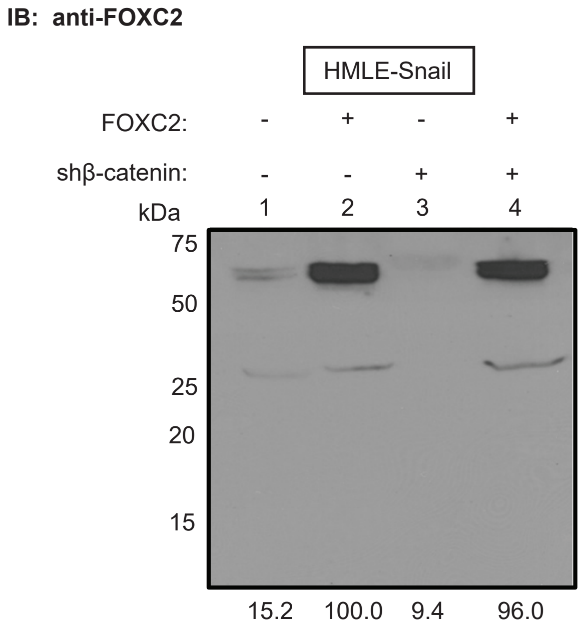
IB: anti-FOXC2
HMLE-Snail
FOXC2: - + - +
shβ-catenin: - - + +
kDa 1 2 3 4
75
50
25
20
15
15.2 100.0 9.4 96.0

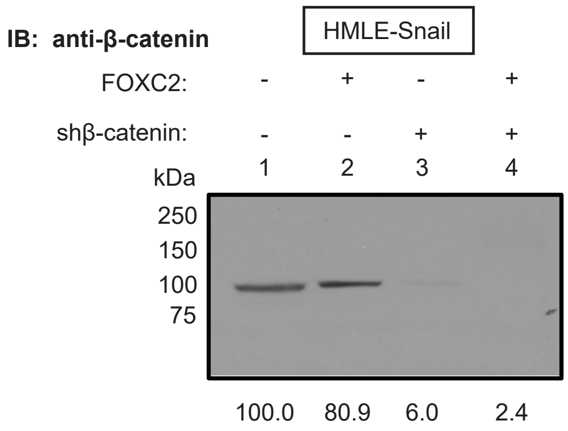
IB: anti-β-catenin
HMLE-Snail
FOXC2: - + - +
shβ-catenin: - - + +
kDa 1 2 3 4
250
150
100
75
100.0 80.9 6.0 2.4

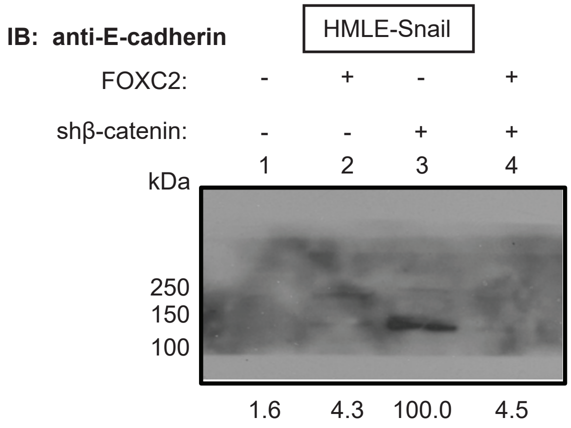
IB: anti-E-cadherin
HMLE-Snail
FOXC2: - + - +
shβ-catenin: - - + +
kDa 1 2 3 4
250
150
100
1.6 4.3 100.0 4.5

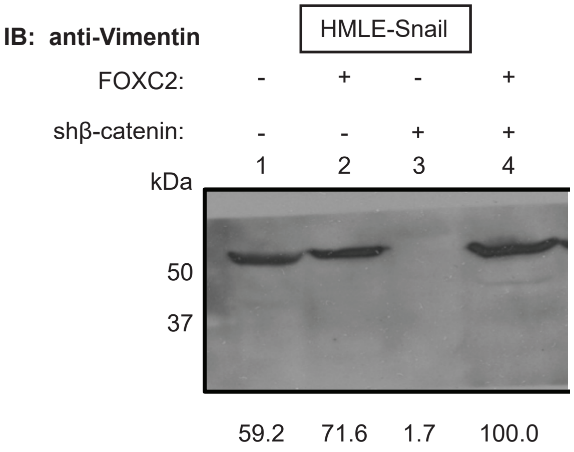
IB: anti-Vimentin
HMLE-Snail
FOXC2: - + - +
shβ-catenin: - - + +
1 2 3 4
kDa
50
37
59.2 71.6 1.7 100.0

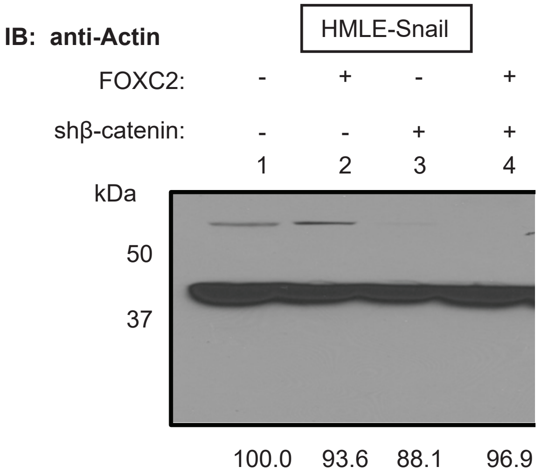
IB: anti-Actin
HMLE-Snail
FOXC2: - + - +
shβ-catenin: - - + +
1 2 3 4
kDa
50
37
100.0 93.6 88.1 96.9

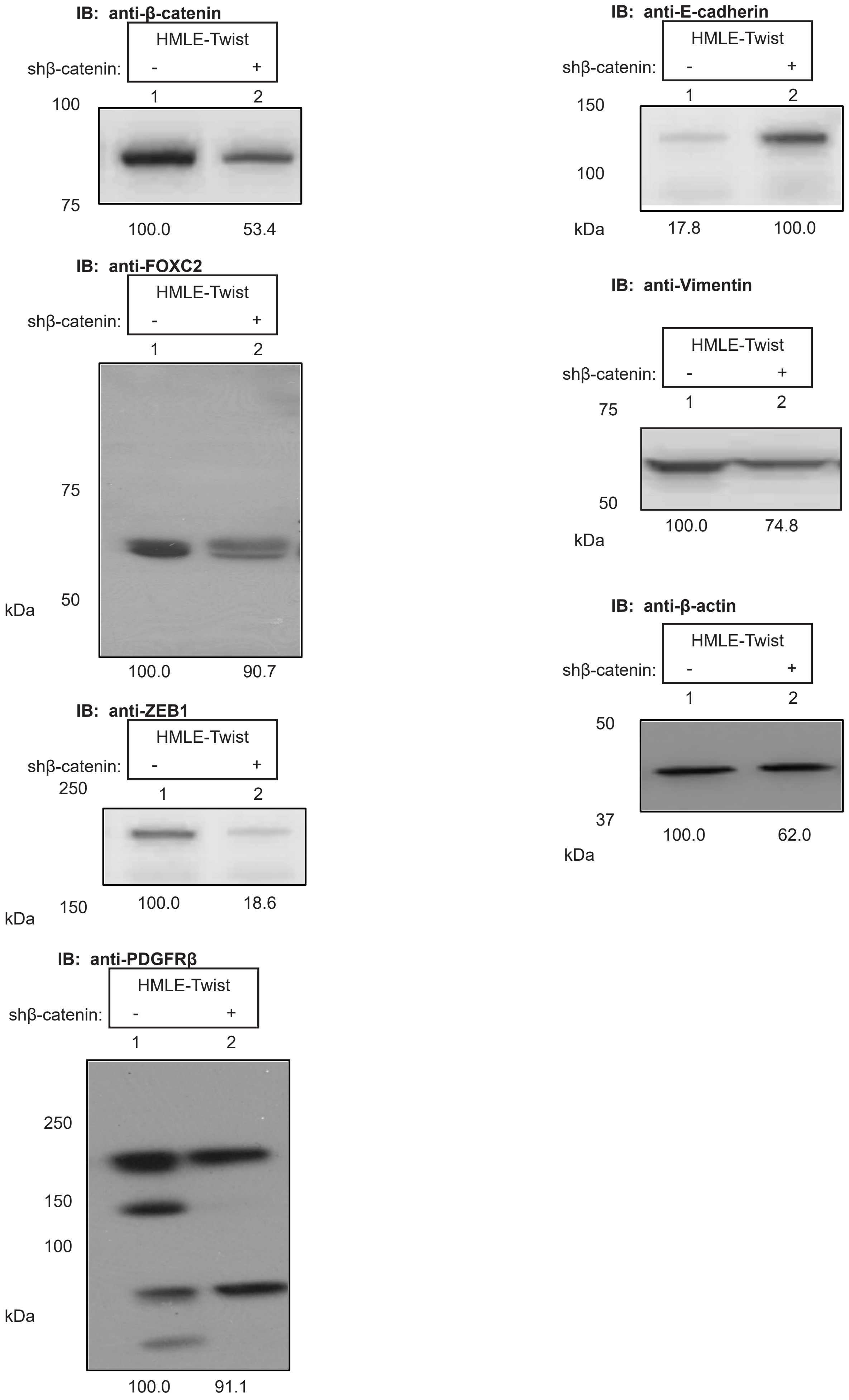
IB: anti-β-catenin
HMLE-Twist
shβ-catenin: - +
1 2
100
75
100.0 53.4
IB: anti-FOXC2
HMLE-Twist
shβ-catenin: - +
1 2
75
50
kDa
100.0 90.7
IB: anti-ZEB1
HMLE-Twist
shβ-catenin: - +
1 2
250
150
kDa
100.0 18.6
IB: anti-PDGFRβ
HMLE-Twist
shβ-catenin: - +
1 2
250
150
100
kDa
100.0 91.1
IB: anti-E-cadherin
HMLE-Twist
shβ-catenin: - +
1 2
150
100
kDa
17.8 100.0
IB: anti-Vimentin
HMLE-Twist
shβ-catenin: - +
1 2
75
50
kDa
100.0 74.8
IB: anti-β-actin
HMLE-Twist
shβ-catenin: - +
1 2
50
37
kDa
100.0 62.0

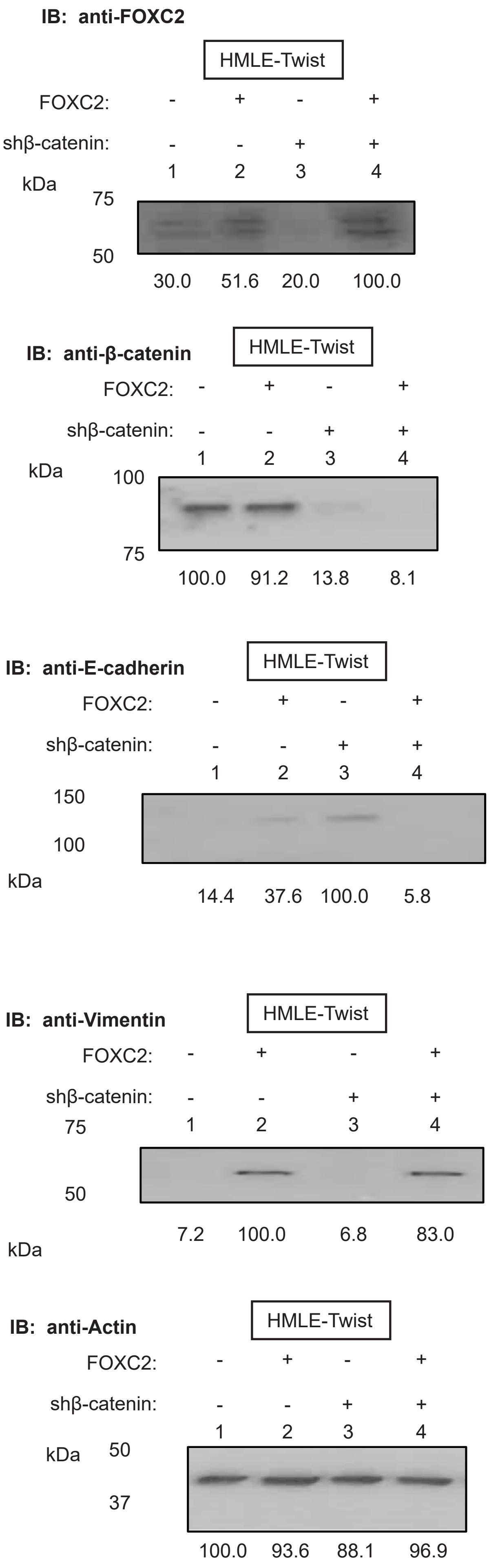
IB: anti-FOXC2
HMLE-Twist
FOXC2: - + - +
shβ-catenin: - - + +
1 2 3 4
kDa
75
50
30.0 51.6 20.0 100.0
IB: anti-β-catenin
HMLE-Twist
FOXC2: - + - +
shβ-catenin: - - + +
1 2 3 4
kDa
100
75
100.0 91.2 13.8 8.1
IB: anti-E-cadherin
HMLE-Twist
FOXC2: - + - +
shβ-catenin: - - + +
1 2 3 4
150
100
kDa
14.4 37.6 100.0 5.8
IB: anti-Vimentin
HMLE-Twist
FOXC2: - + - +
shβ-catenin: - - + +
75
1 2 3 4
50
7.2 100.0 6.8 83.0
kDa
IB: anti-Actin
HMLE-Twist
FOXC2: - + - +
shβ-catenin: - - + +
1 2 3 4
kDa
50
37
100.0 93.6 88.1 96.9
